# Barriers and Facilitators in Diagnostic Pathways That Align Universal Tumor Screening and Mainstream Genetic Testing for Lynch Syndrome in Colorectal Cancer: Protocol for a Scoping Review With a Narrative Synthesis

**DOI:** 10.2196/70831

**Published:** 2025-06-10

**Authors:** Linda Battistuzzi, Eva Blondeaux, Alberto Puccini, Luca Boni, Federica Grillo, Lucia Trevisan, Liliana Varesco, Maria Stefania Sciallero

**Affiliations:** 1 Medical Oncology Unit 2 IRCCS Ospedale Policlinico San Martino Genova Italy; 2 Clinical Epidemiology Unit IRCCS Ospedale Policlinico San Martino Genova Italy; 3 Department of Biomedical Sciences Humanitas University Milan Italy; 4 Medical Oncology and Hematology Unit IRCCS Humanitas Research Hospital Humanitas Cancer Center Milan Italy; 5 Anatomic Pathology Unit IRCCS Ospedale Policlinico San Martino Genova Italy; 6 Department of Surgical and Integrated Diagnostic Sciences University of Genova Genova Italy; 7 Hereditary Cancer Unit IRCCS Ospedale Policlinico San Martino Genova Italy; 8 Medical Oncology Unit 1 IRCCS Ospedale Policlinico San Martino Genova Italy

**Keywords:** Scoping review, protocol, colorectal cancer, Lynch syndrome, diagnostic pathway, genetic testing, mainstreaming, barriers, facilitators, implementation, theoretical domains framework

## Abstract

**Background:**

Approximately 3% of colorectal cancers (CRCs) are due to Lynch syndrome (LS), a hereditary cancer syndrome caused by pathogenic variants (PVs) in the mismatch repair (MMR) genes. Patients with CRC and LS have elevated lifetime risks for a range of cancers and require personalized treatment and targeted surveillance. Relatives of people affected by LS who share the same PV also have elevated cancer risks and can benefit from preventive measures and/or risk-reducing surgeries. Despite this, LS remains vastly underdiagnosed. Universal tumor screening (UTS) for deficient MMR is recommended in diagnosing LS in patients with CRC. This process, when combined with genetic testing (GT) offered within routine cancer care (termed “mainstream GT”), aims to identify individuals at risk efficiently, but integrating UTS and mainstream GT for LS in CRC is a complex endeavor.

**Objective:**

The aim of the proposed scoping review will be to comprehensively explore the literature on diagnostic pathways comprising UTS and mainstream GT for LS among patients with CRC and barriers and facilitators in their implementation.

**Methods:**

The scoping review will follow Arksey and O’Malley’s expanded framework. Results will be reported following the PRISMA-ScR (Preferred Reporting Items for Systematic Reviews and Meta-Analyses extension for Scoping Reviews) guidelines and summarized quantitatively. A narrative synthesis will also be performed using the Theoretical Domains Framework.

**Results:**

The results will be presented in a forthcoming scoping review, which we expect to publish in a peer-reviewed journal by early 2026.

**Conclusions:**

Aligning UTS with mainstream GT for LS in CRC may boost early diagnosis and prevention while reducing waiting times and other patient burdens. By addressing barriers to and facilitators in diagnostic pathways, health care systems can improve the identification and management of LS, ultimately leading to better outcomes for patients and their families. The insights gained from this scoping review will inform the development of a mixed methods study about implementing diagnostic pathways for LS in CRC that integrate UTS and mainstream GT in Italy.

**International Registered Report Identifier (IRRID):**

PRR1-10.2196/70831

## Introduction

### Background

Colorectal cancer (CRC) is the third-most prevalent cancer among men and the second-most prevalent cancer among women globally [1]. Approximately 3% of CRCs are due to Lynch syndrome (LS), an autosomal dominant hereditary cancer syndrome caused by pathogenic variants (PVs) in the mismatch repair (MMR) genes *MLH1, MSH2, MSH6, PMS2*, or *EPCAM*, which are related to the microsatellite instability (MSI) system [2].

Individuals with LS have elevated lifetime risks for CRC and endometrial carcinoma [3]. Risks for other cancers, such as ovarian, stomach, small bowel, urothelial, pancreaticobiliary, and brain cancers, are also greater than for the general population [4,5]. Patients with CRC with LS require personalized treatment and targeted surveillance for metachronous primary CRCs and other LS-associated cancers [6]. Furthermore, as each child of a PV carrier has a 50% chance of inheriting the PV, many of the bloodline relatives of patients with CRC with LS will also have inherited the LS-associated PV and, therefore, the associated cancer risks. Identifying these at-risk relatives can translate into opportunities to prevent CRC, endometrial, and other LS-associated cancers through intensive surveillance and/or risk-reducing surgeries [7].

### Challenges in the Diagnosis of LS

Despite these widely demonstrated clinical benefits, most patients with CRC who are affected by LS are never diagnosed with the syndrome, a failure that has long been acknowledged [8].

Previously, access to genetic testing (GT) for LS was granted only through familial and clinical criteria [9]. These criteria have been shown to miss as many as 25% of patients with LS [10], likely because of the complexity of the criteria and the fact that family histories are often unavailable or inaccurate.

To circumvent such limitations, universal screening of all CRC tumors for deficient MMR at the point of diagnosis (universal tumor screening [UTS]) has been endorsed as the standard of care by multiple societies [11-13]. The diagnostic process includes tumor analysis for the loss of MSH2, MSH6, or PMS2 expression, which, if identified, is an indication for genetic counseling and germline GT. Loss of MLH1 expression during immunohistochemistry may be due to promotor methylation with somatic loss (about 80% of cases with loss of MLH14 expression) or less frequently following germline mutation. CRCs with somatic loss of MLH1 (approximately 10%-15%) [14] are excluded by reflex testing for the *BRAF* mutation or *MLH1* promoter methylation prior to patient referral for genetic counseling and testing [13,15].

This screening process involves multiple steps with coordination across multiple departments, which may be logistically complex and costly, and compete with other priorities [10,16,17]. Thus, it is perhaps not surprising that the uptake of UTS-based testing guidelines for LS has been low [18-20].

### New Opportunities to Diagnose LS in Patients With CRC

A new impetus for the identification of LS in patients with CRC has arisen from the emergence of immunotherapy based on PD-L1 (programmed cell death ligand 1) blockade, with pembrolizumab being the first site-agnostic agent to be licensed for cancer treatment. This has made the identification of MMR-deficient tumors, whether sporadic or hereditary [21], a growing need in patient care and is generating new opportunities to improve the identification of patients with CRC with LS and their at-risk relatives [22]. More generally, it has promoted the integration of GT for LS into the routine care of patients with CRC [19], an approach termed the mainstreaming of GT [23]. The new algorithm involves performing UTS for deficient MMR protein function analysis alongside reflex testing for *BRAF* and/or *MLH1* promoter methylation to exclude sporadic cases and then directing selected patients to GT through the oncologist (or another specialist health care professional) instead of via referral to genetic services.

### The Need to Better Understand Barriers and Facilitators in New Diagnostic Pathways for LS

International studies that reported challenges in implementing UTS [24,25] and mainstream GT in hereditary cancer [26-28] can provide valuable lessons on how to align UTS with mainstream GT. However, diagnostic pathways that integrate UTS and mainstream GT for LS in CRC may vary, as they can, for instance, involve different approaches to pretest counseling and informed consent, with GT being offered by a range of nongenetic health care professionals with different levels of familiarity with genetics and different training needs [27,29]. Variability can also occur in terms of how testing is promoted, staff is educated and trained, appointments are streamlined, and workflows are organized [30] and therefore requires specific attention, with a view to understanding what specific barriers and facilitators can exist in the context of multistakeholder implementation of these novel diagnostic pathways [23,31]. Therefore, we intend to conduct a scoping review that will identify, summarize, and synthesize knowledge about diagnostic pathways that integrate UTS and mainstream GT for LS among patients with CRC and the barriers and facilitators in their implementation.

## Methods

### Overview

The proposed scoping review will be based on the well-established framework developed by Arksey and O'Malley [32] and on the recommendations later proposed by Colquhoun et al [33] and Levac et al [34]. The findings will be reported as recommended by the PRISMA-ScR (Preferred Reporting Items for Systematic Reviews and Meta-Analysis extension for Scoping Reviews) guidelines.

A scoping review is a thorough, rigorous, transparent tool for knowledge synthesis that maps an existing or emerging body of literature to provide an overview of the size and scope of a research field [34].

Scoping reviews are especially appropriate when evidence on a topic is emerging or when the relevant literature is complex or heterogeneous. Furthermore, they can examine findings from different study designs and methods [34]. Therefore, scoping reviews are ideal for synthesizing literature on topics that still need to be comprehensively explored, such as the one we will endeavor to cover.

### Stage 1: Identifying the Research Questions

The research questions of the proposed scoping review are as follows:

1. Which are the diagnostic pathways for LS that align UTS with mainstream GT for LS in CRC described in the literature?
2. What has been reported about the barriers and facilitators in the implementation of these diagnostic pathways?
3. What are the knowledge gaps regarding the barriers and facilitators in the implementation of these diagnostic pathways?

An exploratory search in PROSPERO, the International Prospective Register of Systematic Reviews of the National Institute for Health Research, and the Cochrane Library revealed no comprehensive systematic reviews addressing similar questions. In addition, no previous scoping reviews of the literature on this topic were found.

The research team will revise the protocol as appropriate during the review process. Any changes to the protocol will be detailed and justified in the final report.

### Stage 2: Identifying Relevant Studies

#### Eligibility Criteria

To determine the eligibility criteria of the articles to be included in the review, the research team will follow the PCC (Population, Concept, Context) framework for scoping reviews by the Joanna Briggs Institute [35]. The inclusion criteria will be as follows.

#### Population

We will include patients with CRC who are older than 18 years and health professionals including pathologists, molecular biologists, gastroenterologists, oncologists, oncology nurses, geneticists, genetic counselors, and surgeons.

#### Concept

We will examine diagnostic pathways that align UTS with mainstream GT for LS in patients with CRC and barriers and facilitators in the implementation of those diagnostic pathways.

#### Context

The study will focus on health care settings. There will be no geographic criteria as we aim to provide a comprehensive examination of the literature on this topic.

#### Other Inclusion Criteria

The following additional inclusion criteria will be considered: articles published in a peer-reviewed outlet, those published in English, those that include the relevant search terms, those in which barriers and facilitators in diagnostic pathways that integrate UTS and mainstream GT for LS in CRC are the main focus or are at least addressed in their own part or section, and those that were published in 2017 or later, after the publication of the relevant NICE (National Institute for Health and Care Excellence) guidelines on molecular testing for LS in people with CRC [13].

#### Exclusion Criteria

We will exclude articles that do not address the integration of UTS and mainstream GT, those that do not focus on patients with CRC, and those that do not describe barriers and facilitators.

#### Search Strategy and Information Sources

The literature search strategy will be developed using MeSH (Medical Subject Headings) terms and keywords related to the research questions. Studies will be identified through searches in MEDLINE, Scopus, CINAHL, and PsycINFO. A specialist librarian will review the search strategy using the Peer Review of Electronic Search Strategies (PRESS) guidelines [36].

Backward and forward reference searches for all eligible articles will then be performed to ensure comprehensive literature coverage. Input from expert collaborators will also be sought to supplement the database searches.

### Stage 3: Selecting the Studies

All results retrieved from the databases will be imported into the reference management software Rayyan [37], and duplicate entries will be removed. In the first screening stage, 2 researchers will independently review the titles and abstracts to assess eligibility. Any disagreements will be discussed until a consensus is reached, involving other coauthors if necessary. Irrelevant articles, theses, articles from the popular press, reports, nonreviewed books and book chapters, presentations, and opinion pieces will be excluded. The second eligibility screening will be conducted independently by the 2 researchers, who will evaluate the full-text articles against the inclusion and exclusion criteria. Any discordances will be discussed, and a consensus will be reached by consulting other coauthors, as appropriate. A PRISMA-ScR flowchart will be developed to document the process.

### Stage 4: Charting the Data

A descriptive quantitative synthesis and a narrative synthesis will be conducted to chart the data. In accordance with Arksey and O’Malley [32], the descriptive quantitative summary will include the final number of articles and key characteristics such as the first author, publication year, country, discipline, and study design (eg, qualitative, quantitative, and mixed methods). This information will be presented in the scoping review report to provide an overview of the included literature.

In the narrative synthesis, the Theoretical Domains Framework (TDF) will be used to guide the analysis of barriers and facilitators and map them to higher-level behavioral domains and components [38]. The TDF is a validated, comprehensive framework consisting of 14 domains that can influence the behavior of health care professionals (knowledge; skills; social/professional role and identity; beliefs about capabilities; optimism; beliefs about consequences; reinforcement; intentions; goals; memory, attention, and decision processes; environmental context and resources; social influences; emotion; and behavioral regulation) and can be adapted to specific clinical contexts [39].

A data extraction tool will be created, incorporating the specific methodological and design aspects of each publication, the study setting, and factors affecting the implementation of new diagnostic pathways for LS. The tool will first be tested on 2 randomly chosen publications, refined as needed, and finalized for use with the remaining studies. Two researchers will independently conduct data extraction, following a predefined codebook that will be continuously updated throughout the extraction and analysis process. The extracted data items will then be grouped into themes. If the data item cannot be matched to an existing theme, a new theme will be created. Each theme will be mapped to a TDF domain, and the frequency of each domain will be calculated as a percentage of the total number of articles. The differences across health care professionals and clinical disciplines will be described narratively [27].

### Stage 5: Collating, Summarizing, and Reporting the Results

The essential characteristics of the studies will be presented in a table format, providing an overall summary of the articles selected. Other results will be presented in diagrams and descriptive summaries as most appropriate to display and convey the extracted information.

## Results

The results will be presented in a forthcoming scoping review, which we expect to publish in a peer-reviewed journal by early 2026.

## Discussion

Diagnostic pathways standardize diagnostic approaches, enabling equitable access to evidence-based care [40]. In LS, aligning UTS with mainstream GT in patients with CRC may boost early diagnosis and prevention while at the same time reducing waiting times and other patient burdens. Implementing novel practices in real-world health care settings, however, is challenging, and ensuring adequate uptake of complex interventions such as diagnostic pathways requires putting in place targeted approaches to modify clinical practice behaviors [41].

To the best of our knowledge, the proposed scoping review will be the first systematic effort aimed at mapping the literature on different diagnostic pathways that align UTS and mainstream GT for LS among patients with CRC, along with the barriers and facilitators in their implementation. Its results will inform the development of a mixed methods study to be conducted in Italy, involving pathologists, molecular biologists, gastroenterologists, oncologists, geneticists, and surgeons, and focusing on implementing an integrated UTS–mainstream GT algorithm for LS among patients with CRC [31].

The planned scoping review will have some limitations. The search will be restricted to publications in English for feasibility reasons, and only peer-reviewed articles will be included. Furthermore, as is the norm for scoping reviews, a quality assessment for the articles included will not be performed.

The main strength of the proposed scoping review is that it will use the PRISMA-ScR methodology, which is robust, standardized, and reproducible. Another strength is that it will rely on a theoretically underpinned implementation framework, TDF, to identify behavior change determinants (barriers and facilitators) [42], support improved understandings of mechanisms of action, and, ultimately, the development of more effective and sustained interventions [31].

## Appendix

Multimedia Appendix 1 PRISMA (Preferred Reporting Items for Systematic Reviews and Meta-Analyses) Scoping Review Checklist.

## Abbreviations

CRC: colorectal cancer
GT: genetic testing
LS: Lynch syndrome
MeSH: Medical Subject Headings
MMR: mismatch repair
MSI: microsatellite instability
NICE: National Institute for Health and Care Excellence
PCC: Population, Concept, Context
PD-L1: programmed cell death ligand 1
PRESS: Peer Review of Electronic Search Strategies
PRISMA-ScR: Preferred Reporting Items for Systematic Reviews and Meta-Analyses extension for Scoping Reviews
PV: pathogenic variant
TDF: Theoretical Domains Framework
UTS: universal tumor screening
